# Efficacy of Dengue Vaccines in the Prevention of Severe Dengue in Children: A Systematic Review

**DOI:** 10.7759/cureus.28916

**Published:** 2022-09-07

**Authors:** Paul Foucambert, Faith D Esbrand, Sana Zafar, Venkatesh Panthangi, Adrienne R Cyril Kurupp, Anjumol Raju, Gaurav Luthra, Mahrukh Shahbaz, Halah Almatooq, Safeera Khan

**Affiliations:** 1 Internal Medicine, California Institute of Behavioral Neurosciences & Psychology, Fairfield, USA; 2 Pediatrics, California Institute of Behavioral Neurosciences & Psychology, Fairfield, USA; 3 Dermatology, California Institute of Behavioral Neurosciences & Psychology, Fairfield, USA

**Keywords:** dengvaxia, dengue vaccination, dengue tetravalent vaccine, dengue vaccine, dengue infection, dengue fever, dengue shock syndrome, dengue hemorrhagic fever, severe dengue, dengue

## Abstract

Dengue is a vector-borne disease caused by the dengue virus (DENV) and is a major health concern worldwide, particularly in regions of endemic disease. Dengue usually presents as a self-limited febrile illness. In some cases, more severe forms with hemorrhage and shock can occur, and children are especially prone to develop it. These forms can be lethal without appropriate management, and no antiviral treatment exists today. In the absence of a curative treatment for dengue, its clinical prevention remains essential. One vaccine - the chimeric yellow fever-dengue-tetravalent dengue vaccine (CYD-TDV) - has been approved for use in some populations, and several others are currently in development, including Takeda's tetravalent dengue vaccine candidate (TAK-003).

This study is a systematic review of the current literature realized to evaluate the efficacy of the dengue vaccines in preventing severe dengue in children. This review focuses on the vaccines CYD-TDV and TAK-003. This systematic review was conducted according to the Preferred Reporting Items for Systematic Reviews and Meta-Analyses (PRISMA) guidelines. PubMed, PubMed Central (PMC), Medical Literature Analysis and Retrieval System Online (MEDLINE), Cochrane Library, and Google Scholar were the databases used to find the relevant data. The articles were selected using specific inclusion and exclusion criteria, and quality appraisal was realized with standardized quality assessment tools. Overall, our study shows that the dengue vaccines CYD-TDV and TAK-003 confer protection against severe dengue in children. Some distinctions exist depending on the vaccine type, the age, and the dengue serostatus of patients. While demonstrating encouraging results, this review also emphasizes the need for more in-depth studies about the safety and efficacy of dengue vaccines.

## Introduction and background

Dengue incidence has increased 30 times in the last 50 years [1]. Nowadays, 2.5 billion people live in endemic areas, and it is estimated that 50 to 100 million infections occur worldwide, resulting in 25000 deaths [1,2]. Dengue is the most prevalent arthropod-borne viral disease worldwide [1]. It is endemic in more than 100 countries in tropical and subtropical regions of Southeast Asia, Africa, the West Pacific, and Central and South America [1,2]. Dengue is also seen in some regions of Europe, including France, Croatia, Portugal, and Germany, and some parts of the United States (US), such as Hawaii, Florida, and the US Virgin Islands [3].

Dengue is caused by the dengue virus (DENV), a flavivirus of the family Flaviviridae, and is transmitted to humans by the *Aedes aegypti* mosquito. It has four major serotypes: DENV1, DENV2, DENV3, and DENV4 [4]. Infection with dengue virus has a wide variety of presentations, ranging from asymptomatic to severe forms, including hemorrhage and shock. Most dengue infections present with dengue fever (DF), an acute febrile illness characterized by fever, myalgias, arthralgias, retro-orbital pain, maculopapular rash, lymphadenopathy, lymphopenia, and thrombocytopenia [5]. Infected children are likely to present with a mild febrile illness and a rash. DF is self-limited and usually lasts five to seven days [6].

In some cases, dengue infection can lead to dengue hemorrhagic fever (DHF) or dengue shock syndrome (DSS), which is usually present in children aged less than 15 years [7]. DHF is characterized by thrombocytopenia, fever, hemorrhage, and plasma leakage and usually lasts seven to 10 days [4]. When plasma leakage becomes critical, DSS can ensue. It is a form of hypovolemic shock causing decreased tissue perfusion and multi-organ failure [5,6]. More than 500000 cases of DHF and DSS occur annually and are associated with 1% to 10% fatality rates, which may be as high as 30% without adequate management [7]. In 2009, the World Health Organization (WHO) reclassified dengue severity into dengue with or without warning signs and severe dengue. Severe dengue includes dengue with severe plasma leakage, severe bleeding, and/or severe organ involvement [8].

There is currently no treatment for dengue infection, and the management consists mainly of supportive care [9]. The clinical prevention of dengue has recently become possible through the development of vaccines. The first licensed dengue vaccine was the chimeric yellow fever-dengue-tetravalent dengue vaccine (CYD-TDV) Dengvaxia® from Sanofi Pasteur, Paris, France, in 2015, which is now approved for use in 19 countries [10]. However, its protective efficacy was lower against the serotype DENV2 than other serotypes. A lower efficacy was also noted in children younger than nine and dengue-naïve subjects [11]. A risk of vaccine-exacerbated disease was raised, and in 2018, the vaccine was recommended for use in people aged nine years and more with a history of previous dengue infection [12]. Other candidate vaccines are currently in development. The National Institute of Allergy and Infectious Diseases (NIAID) is developing the dengue live attenuated tetravalent vaccines TV003 and TV005, which are undergoing phase 2 and 3 clinical trials [11,13]. Another new tetravalent vaccine candidate is in development: Takeda's tetravalent dengue vaccine candidate (TAK-003). It is based on a live attenuated DENV2 virus and is currently undergoing phase 3 clinical trials [14].

In the absence of curative treatment and with a significantly high number of severe cases in children every year, the need for a review to evaluate our current capacity to prevent severe infection in this population was clear to us. Therefore, we will be conducting a systematic review of the current literature to assess the efficacy of the different dengue vaccines in preventing severe dengue infection and DHF in populations aged zero to 18 years.

## Review

Methods

We conducted this systematic review according to the Preferred Reporting Items for Systematic Reviews and Meta-Analyses (PRISMA) guidelines. The results were reported in line with these standards and principles [15]. This research was realized in April and May 2022.

Search Strategy

On April 4, 2022, we used the databases PubMed, PubMed Central (PMC), Medical Literature Analysis and Retrieval System Online (MEDLINE), Cochrane Library, and Google Scholar to extract the articles relevant to this review. To conduct our search on PubMed, we used the regular search tool. We looked for the keywords "Dengue fever," "Dengue infection," "Dengue," "Dengue vaccine," "Dengue vaccination," "Dengue tetravalent vaccine," "Dengvaxia," "Dengue hemorrhagic fever," "Dengue shock syndrome," and "severe dengue." The search was (Dengue fever OR Dengue infection OR Dengue) AND (Dengue vaccine OR Dengue vaccination OR Dengue tetravalent vaccine OR Dengvaxia) AND (Dengue hemorrhagic fever OR Dengue shock syndrome OR Severe dengue). We applied the following filters: studies from the last 10 years, human studies, studies in the English language, children subjects (0-18 years), and full-text articles. We also used the Medical Subject Heading (MeSH) strategy with the following combination of keywords: ("Dengue/complications" {Majr} OR "Dengue/drug therapy" {Majr} OR "Dengue/mortality" {Majr} OR "Dengue/prevention and control" {Majr} OR "Dengue/statistics and numerical data" {Majr}) AND ("Dengue Vaccines/pharmacology" {Majr} OR "Dengue Vaccines/statistics and numerical data" {Majr} OR "Dengue Vaccines/therapeutic use" {Majr}) AND ("Severe Dengue/complications" {Majr} OR "Severe Dengue/drug therapy" {Majr} OR "Severe Dengue/prevention and control" {Majr} OR "Severe Dengue/statistics and numerical data" {Majr}). We applied the same filters as stated previously. We used the same combination of keywords to conduct our searches on Cochrane Library and Google Scholar and looked for published articles in the last 10 years.

Inclusion and Exclusion Criteria

We used the following criteria to select the articles included in this review. Criteria were decided before searching for articles. Severe dengue is a term that emerged from the new classification of dengue severity by the WHO in 2009. The previous classification from 1997 categorized dengue into dengue fever, dengue hemorrhagic fever, and dengue shock syndrome [8]. The studies we collected reported severe dengue cases, cases of DHF, or neither. We considered that selecting cases only based on one classification would exclude relevant data; accordingly, we decided to include cases of severe dengue and DHF/DSS, considering criteria from 1997 and 2009.

Inclusion criteria were articles related to our topic, peer-reviewed articles, publications from the last 10 years, studies in the English language, human studies, children subjects (0-18 years), original studies, and long-term follow-ups. Exclusion criteria were grey literature, not peer-reviewed articles, unpublished literature, review articles, and secondary analyses.

Study Selection

Two authors (PF and VP) conducted the screening of articles independently. The first screening step was done by automation filter tools on the different databases. Next, the reviewers removed duplicate articles extracted from PubMed using the EndNote citation manager and removed the duplicate articles from other databases manually. Articles were screened based on their titles and abstracts and reading full texts. Articles were selected using the eligibility criteria stated previously. The quality of articles was then checked using standardized quality assessment tools.

Quality Assessment

Two authors (PF and VP) realized the quality assessment of the studies independently using the revised Cochrane risk-of-bias tool for randomized trials (RoB 2) and the Joanna Briggs Institute (JBI) risk assessment tool for case-control. For the Revised Cochrane RoB 2 tool, we used the signs "+," "-," and "?" that indicate high risk of bias, low risk of bias, and unclear risk of bias, respectively. We decided that a minimum of six "+" would be required to be considered high quality and included in our review. When using the JBI risk assessment tool, we chose the words "Yes," "No," and "Unclear" to answer the different questions. A minimum of eight "Yes" was required to consider the study of high quality and retain it in our review.

Results

Search Results and Selection of Articles

**Figure 1:**
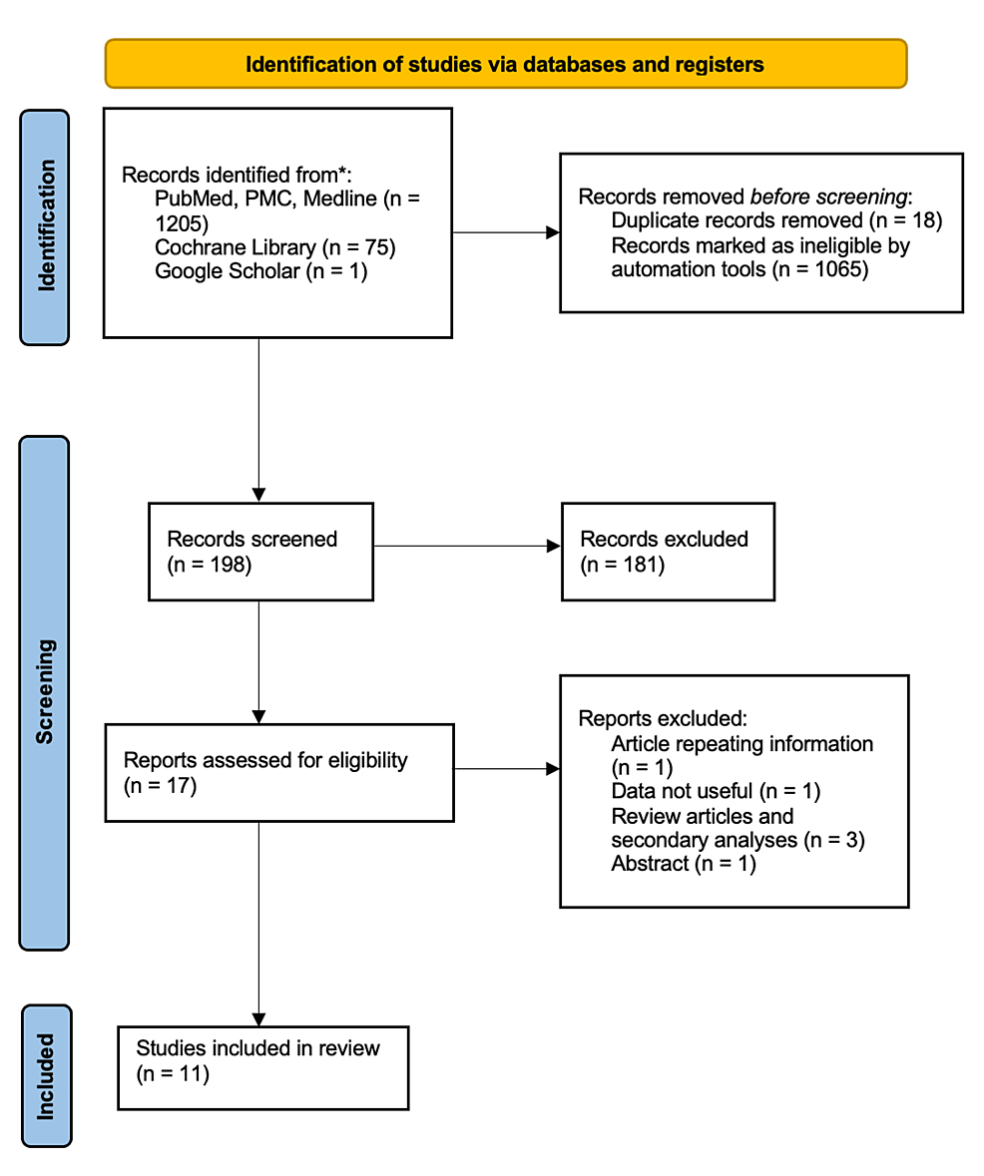
PRISMA flow diagram PMC: PubMed Central, PRISMA: Preferred Reporting Items for Systematic Reviews and Meta-Analyses, MEDLINE: Medical Literature Analysis and Retrieval System Online

Our search using keywords on PubMed, PMC, and MEDLINE resulted in 1200 articles. After the application of the filters, 136 articles were left. Our search using MeSH keywords resulted in five articles. After the application of the same filters, four articles were left. Our search on Cochrane Library resulted in 75 articles. We also added one relevant study from Google Scholar that was not present in our initial list of articles. The combined result after searching all databases was 216 articles. Out of those 216 articles, we removed 18 duplicates. We screened the 198 articles by reading titles and abstracts and then removed 181 articles. Out of the 17 articles left, one had information already present in other articles, one was reporting data that we considered not valuable to our study, three were review articles, and one was an abstract. This led to a final number of 11 relevant articles. This sequence of events is illustrated in the PRISMA flow diagram (Figure 1).

Quality of Studies

After assessing 10 randomized controlled trials (RCTs) for quality, we attributed six "+" to four of them and seven "+" to six. We considered these studies high quality and decided to include them in our systematic review. The results are exposed in Table 1.

**Table 1:** Quality assessment of RCTs RCTs: randomized controlled trials Capeding et al., 2014 [16]; Villar et al., 2015 [17]; Sabchareon et al., 2012 [18]; Limkittikul et al., 2019 [19]; Biswal et al., 2020 [20]; Lanata et al., 2012 [21]; Tricou et al., 2020 [22]; Tran et al., 2012 [23]; Arredondo-García et al., 2018 [24]; Forrat et al., 2021 [25]

Studies	Random sequence generation (selection bias)	Allocation concealment (selection bias)	Blinding of participants	Blinding of personnel/care providers (performance bias)	Blinding of outcome assessor (detection bias)	Incomplete outcome data (attrition bias)	Selective reporting (reporting bias)	Other biases	Overall
Capeding et al., 2014 [16]	+	+	+	+	+	+	+	-	7/8
Villar et al., 2015 [17]	+	+	+	+	+	+	+	-	7/8
Sabchareon et al., 2012 [18]	+	+	+	+	-	+	+	-	6/8
Limkittikul et al., 2019 [19]	+	+	+	+	-	+	+	-	6/8
Biswal et al., 2020 [20]	+	+	+	+	+	+	+	-	7/8
Lanata et al., 2012 [21]	+	+	+	+	+	+	+	-	7/8
Tricou et al., 2020 [22]	+	+	+	+	+	+	+	-	7/8
Tran et al., 2012 [23]	+	+	+	+	+	+	+	-	7/8
Arredondo-García et al., 2018 [24]	+	+	+	+	?	+	+	-	6/8
Forrat et al., 2021 [25]	+	+	+	+	?	+	+	-	6/8

We checked for the quality of one case-control study and assigned nine "Yes." We only considered that published research with high quality and decided to include it in our systematic review. The results are shown in Table 2.

**Table 2:** Quality assessment of case-control study Ylade et al., 2021 [26]

Study	Were the groups compared to the presence of disease in cases and the absence of disease in controls?	Were cases and controls matched appropriately?	Were the same criteria used for the identification of cases and controls?	Was exposure measured in a standard, valid, and reliable way?	Was exposure measured in the same way for cases and controls?	Were confounding factors identified?	Were strategies to deal with confounding factors stated?	Were outcomes assessed in a standard, valid, and reliable way for cases and controls?	Was the exposure period of interest long enough to be meaningful?	Was appropriate statistical analysis used?	Overall appraisal
Ylade et al., 2021 [26]	Yes	Yes	Yes	Yes	Yes	Unclear	Yes	Yes	Yes	Yes	Include

Study Characteristics

Out of 11 articles, 10 were randomized controlled trials (RCTs) or their follow-up, and one was a case-control study. Six studies focused on efficacy, three studies focused on safety and immunogenicity, one study focused on safety and efficacy, and one study evaluated all three characteristics of the vaccine. Of the 11 studies, nine evaluated the dengue vaccine CYD-TDV from Sanofi Pasteur, and two evaluated the dengue vaccine TAK-003 from Takeda. The characteristics, results, and conclusions of each study are summarized in Table 3.

**Table 3:** Characteristics, results, and conclusions of selected studies CYD-TDV: chimeric yellow fever-dengue-tetravalent dengue vaccine; NA: not applicable; RCT: randomized controlled trial; TAK-003: Takeda's tetravalent dengue vaccine candidate Forrat et al., 2021 [25]; Ylade et al., 2021 [26]; Biswal et al., 2020 [20]; Tricou et al., 2020 [22]; Limkittikul et al., 2019 [19]; Arredondo-García et al., 2018 [24]; Villar et al., 2015 [17]; Capeding et al., 2014 [16]; Tran et al., 2012 [23]; Sabchareon et al., 2012 [18]; Lanata et al., 2012 [21]

Author and year of publication	Purpose	Study type	Number of participants	Age of participants	Study duration	Results and conclusion regarding the efficacy of the vaccine against severe dengue and/or dengue hemorrhagic fever (DHF)
1. Forrat et al., 2021 [25]	Six-year follow-up of the CYD-TDV vaccine efficacy trials with an evaluation of hospitalized and severe cases by dengue serologic status and focusing on dengue-seropositive individuals	RCTs	29229 (9874 participants from the study CYD14 [16], 16319 participants from the study CYD15 [17], and 3036 participants from the study CYD57 [19])	2-16 years	Six years	In seropositive individuals aged nine years and older, the risk of hospitalized and severe virologically confirmed dengue (VCD) was significantly lower in the CYD-TDV group than in the placebo group over six years. In seropositive individuals aged less than nine years, the risk for hospitalized and severe VCD was significantly lower only in years 1-2, and results were not statistically significant anymore from years 3 to 6. The risk of hospitalized and severe VCD was also lower in seropositive participants aged 6-8 years, with significant results in years 1-2 and 5-6 but less precise in years 3-4. In seronegative individuals aged nine years and older, there was a higher risk of hospitalized and severe VCD in the vaccine group than in the placebo group. Still, results were only statistically significant in one subgroup for severe dengue. There was also a higher risk in seronegative participants less than nine years, with statistically significant results in two subgroups for hospitalized dengue.
2. Ylade et al., 2021 [26]	To evaluate the efficacy of a single dose of the CYD-TDV vaccine against hospitalized VCD	Case-control study	1470 (490 cases and 980 controls)	9-17 years	NA	The crude odds ratio (OR) for a single dose of the dengue vaccine CYD-TDV between cases with warning signs/severe dengue and their matched controls was 0.54 (95% CI: 0.36-0.80), and the adjusted OR was 0.52 (95% CI: 0.34-0.80). The vaccine showed an efficacy of 48% (95% CI: 0.20-0.66) against dengue with warning signs combined with severe dengue.
3. Biswal et al., 2020 [20]	To evaluate the efficacy, safety, and immunogenicity of two doses of the TAK-003 vaccine in healthy children	RCT	20099 (13401 in the vaccine group and 6698 in the control group)	4-16 years	18 months	Two cases of dengue hemorrhagic fever (DHF) occurred in the vaccine group, and seven cases of DHF occurred in the control group. The efficacy of the TAK-003 vaccine against DHF was 85.9% (95% CI: 31.9-97.11). Two cases of severe dengue occurred in the vaccine group and one case in the placebo group. One case in the vaccine group met the WHO criteria for DHF and Dengue Case Severity Adjudication Committee (DCAC) criteria for severe dengue. Three cases in the vaccine group and eight in the placebo group met the criteria for either classification. Overall, no statistically significant efficacy was noted against severe dengue when using only the DCAC classification.
4. Tricou et al., 2020 [22]	To assess the safety and immunogenicity of the TAK-003 vaccine in children living in dengue-endemic regions	RCT	1800 (201 in the two-dose primary series group, 398 in one primary dose group, 1002 in one primary dose + one-year booster group, and 199 in the placebo group)	2-17 years	48 months	Thirty-seven (2%) participants in the vaccine group had VCD, and 13 (7%) in the placebo had VCD during the study period. The relative risk (RR) of VCD in the vaccine receivers was 0.35 (95% CI: 0.19-0.65). No case was considered to be severe dengue.
5. Limkittikul et al., 2019 [19]	Four-year safety follow-up of a CYD-TDV vaccine efficacy trial with an observation of hospitalized VCD cases (CD57)	RCT	3203 (participants from the study CYD23 [18]: 2131 in the vaccine group and 1072 in the control group)	4-11 years	Four years	In the vaccine group, there were 10 cases of severely hospitalized dengue during the study. In the control group, there were five cases of severe hospitalized dengue. It was concluded that there was no significant difference in risk of severe hospitalized dengue between treatment groups.
6. Arredondo-García et al., 2018 [24]	Four-year safety follow-up of two CYD-TDV vaccine efficacy trials with an evaluation of the risk of hospitalized VCD and severe hospitalized VCD	RCTs	35123 (participants from the studies CYD14 [16], CYD15 [17], and CYD57 [19]: 23429 in the vaccine group and 11694 in the control group)	2-16 years	Four years	The cumulative RR in years 1-4 of clinically severe hospitalized VCD was 0.511 in the three studies combined. After stratification by age, the cumulative RR in children aged nine years or more was 0.242. In children less than nine years, the cumulative RR was 1.029. There was considered an overall reduction in the risk of clinically severe hospitalized VCD. Still, no conclusion could be made due to the discrepancy between age groups and variability of results over the years.
7. Villar et al., 2015 [17]	To evaluate the efficacy of the CYD-TDV vaccine against symptomatic VCD after three injections (CD15)	RCT	20869 (13920 in the vaccine group and 6949 in the control group)	9-16 years	25 months	There was one case of severe dengue in the vaccine group and 11 cases in the control group (who received a placebo). The reported efficacy of the CYD-TDV vaccine against severe dengue was 91.7% (95% CI: 31.4-99.8) after three injections.
8. Capeding et al., 2014 [16]	To assess the efficacy of the CYD-TDV vaccine against symptomatic VCD after three doses (CYD14)	RCT	10275 (6851 in the vaccine group and 3424 in the control group)	2-14 years	25 months	In the vaccine group, eight cases of DHF and four clinically severe dengue were not classified as DHF. There were 20 cases of DHF in the placebo group. The CYD-TDV vaccine showed efficacy against DHF/severe dengue of 80.8% (95% CI: 42.7-94.7) after three injections.
9. Tran et al., 2012 [23]	To evaluate the safety and immunogenicity of three doses of the CYD-TDV vaccine	RCT	180 (120 in the vaccine group and 60 in the control group)	2-45 years	Six months	Two cases of virologically confirmed DHF occurred in the placebo group and none in the vaccine group.
10. Sabchareon et al., 2012 [18]	To assess the protective efficacy of three doses of the CYD-TDV vaccine against symptomatic VCD (CD23)	RCT	4002 (2669 in the vaccine group and 1333 in the control group)	4-11 years	25 months	Three cases of severe dengue occurred in the vaccine group. In the control group, two cases of severe dengue occurred, one of them being grade 3 DHF. Other episodes were grade 2 DHF or less severe. Overall, it was considered that there was no excess of severe dengue in the vaccine group.
11. Lanata et al., 2012 [21]	To appraise the safety and immunogenicity of three doses of the CYD-TDV vaccine in children with various yellow fever immune status	RCT	300 (200 in the vaccine group and 100 in the control group)	2-11 years	One month	After three doses, one case of VCD occurred in the vaccine group and three in the placebo group. No cases of severe dengue were noted in either group.

Five of the studies we collected either found that the dengue vaccine was efficient in preventing severe dengue and/or DHF or found a smaller number of severe dengue/DHF cases in the vaccine group compared to the control group. Two studies showed that the dengue vaccine was efficient against severe dengue. Still, in some populations, two studies did not find any difference in severe dengue occurrence between the vaccine and control group, and two did not report any severe dengue cases.

We stratified these results based on vaccine type. Regarding the CYD-TDV vaccine, four studies showed that the vaccine was efficient against severe dengue and/or DHF or found a smaller number of severe dengue and/or DHF in the vaccine group. Two studies showed that the vaccine was efficient but only in some age groups. Two studies did not show differences in results between treatment groups, and one study did not report cases of severe dengue and/or DHF. Concerning the TAK-003 vaccine, one study showed a smaller number of cases of severe dengue and DHF in the vaccine group compared to the control, and one study did not report severe cases or cases of DHF.

Discussion

We conducted this systematic review to assess the efficacy of the different dengue vaccines in preventing severe dengue in children. To our knowledge, no previous systematic review of this topic has been done. This article summarizes the evidence from seven RCTs, three RCT follow-up studies, and one case-control study. We gathered relevant information and reported results and conclusions related to our research question.

We reviewed articles focusing on two vaccines: the dengue vaccine CYD-TDV and the TAK-003 vaccine. The CYD-TDV vaccine has been a source of many controversies, especially concerning its safety in younger populations. Its efficacy has also been shown to be variable, demonstrating different results depending on the ages of patients and their dengue serologic status at baseline [12]. On the other hand, the TAK-003 vaccine, which was recently established and is currently undergoing clinical trials, could become a competitive alternative to the CYD-TDV vaccine if studies show satisfactory results. With this background in mind, we will first focus on the CYD-TDV vaccine and then on the TAK-003 vaccine.

CYD-TDV: An Established Efficacy

The studies by Forrat et al., Ylade et al., Limkittikul et al., Arredondo-García et al., Villar et al., Capeding et al., Tran et al., Sabchareon et al., and Lanata et al. evaluated several features of the dengue vaccine CYD-TDV. The studies by Ylade et al., Villar et al., and Capeding et al. demonstrated that the vaccine was effective against severe diseases [16-19,21,23-26]. The RCT reported by Capeding et al. (CYD14) in 2014 and Villar et al. (CYD15) in 2015 showed an efficacy of 80.8% and 91.7%, respectively, for the prevention of severe dengue and DHF after a three-dose vaccination [16,17]. These findings were significant, and the vaccine demonstrated good protection. More recently, Ylade et al. reported vaccine efficacy of 48% in the prevention of dengue with warning signs combined with severe dengue after participants of the study had received one dose of the vaccine [26], which is 32.8% and 43.7% lower than the efficacy results found by the trials CYD14 and CYD15, respectively.

This substantial difference in results might be due to the fact that RCTs CYD14 and CYD15 evaluated the CYD-TDV vaccine efficacy after participants had received three doses of the vaccine. At the same time, the case-control from Ylade et al. assessed the efficacy of a single-dose vaccination. A single dose of vaccine may have led to a less potent immunity than a three-dose series, therefore conferring less protection against dengue infection with a higher number of severe cases and lower vaccine efficacy.

Moreover, RCTs CYD14 and CYD15 have been conducted using very large populations, with a total of 31144 participants, which is significantly more than the study population of Ylade et al., who evaluated 1470 participants in total. This smaller number of participants might have led to a less powerful result. Moreover, large RCTs with clear results, such as the ones we discussed, are level 1 evidence from Sackett, while case-control studies are level 3 [27]. This enhances the fact that the collected RCTs demonstrated higher efficacy against severe dengue/DHF with greater statistical significance and power than the case-control study from Ylade et al. Nevertheless, these three studies showed solid results and proved that the CYD-TDV vaccine confers protection against severe dengue in children after one or three doses. None of these studies reported issues about the vaccine's safety or recipients' serologic status.

The RCT conducted by Tran et al. demonstrated that cases of virologically confirmed DHF occurred only in the group that received a placebo. In contrast, none occurred in the vaccine group. However, since that study evaluated individuals aged two to 45 years and no precision was made concerning the age of participants who developed DHF, we could not conclude vaccine efficacy in children using that study [23].

Safety Concerns and Restrictions of CYD-TDV

Studies from Forrat et al. and Arredondo-García et al. were follow-ups of the RCTs CYD14, CYD15, and CYD23/CYD57. In 2018, Arredondo-García et al. reported cases of hospitalized virologically confirmed dengue (VCD) and clinically severe hospitalized VCD and demonstrated that overall, the vaccine was efficient against severe hospitalized VCD. After a follow-up period of four years, the CYD-TDV vaccine showed a significantly high protective efficacy in children aged nine years and older, with a cumulative relative risk (RR) of 0.242. However, the cumulative RR in children younger than nine years was found to be 1.029, indicating that being vaccinated slightly increased the risk of developing severe hospitalized VCD. This difference in results was even greater when looking at children aged 2-5. The RR in years 3 and 4 in this age range was found to be 6.449, raising serious concerns about the safety of the CYD-TDV vaccine in that population. However, in those aged 6-8 years, the RR was found to be 0.9, indicating vaccine efficacy against severely hospitalized VCD, although lower than in children aged nine years and more [24].

In September 2018, the CYD-TDV vaccine was recommended for use in individuals aged nine to 45 years who had a previous dengue infection and evidence of seropositivity. This was due to concerns about a risk of vaccine-exacerbated disease in seronegative individuals and a risk of a vaccine-associated severe disease in children younger than nine years. A retrospective analysis the year before showed that seropositive individuals, before being vaccinated, had long-term protection against dengue. Still, participants who were seronegative at baseline and who received the vaccine had an increased risk of severe dengue compared to seronegative non-vaccinated individuals [28]. This validated the finding made by Arredondo-García et al. while also adding precisions about the relationship between dengue serostatus, vaccination, and risk of serious disease.

In 2021, Forrat et al. analyzed the six-year follow-up of the trials CYD14, CYD15, and CYD23/CYD57. After six years, they found that the CYD-TDV vaccine protected against severe and hospitalized VCD in children aged nine years and more who were seropositive. The vaccine also demonstrated good efficacy in seropositive children aged six to eight during most follow-up years [25]. These results seem to confirm the findings made by Arredondo-García et al. in 2018, who demonstrated the efficacy of Dengvaxia® in the same age range. However, the study from Forrat et al. added some precision regarding dengue serostatus and vaccine protection. They showed that seronegative children receiving the vaccine had more risk of hospitalized and severe VCD than unvaccinated children.

Data and results reported by Forrat et al. confirm the concerns that emerged in 2018 about the safety of the CYD-TDV vaccine, which indeed seems unsafe in children who had never been infected with the dengue virus and with negative serologic status. In the absence of the first exposure to dengue, vaccination might mimic a primary infection. A second infection in this context could lead to antibody-dependent enhancement of disease and an increased risk of severe dengue. This was shown by previous studies that proposed secondary dengue infection as a potential risk factor for developing DHF and DSS [29]. The impact of dengue serostatus on the safety and efficacy of the CYD-TDV was reviewed by a study in 2018, which observed an increased risk of hospitalization in vaccinated dengue-naïve children aged 2-16 years and also proposed the possibility of dengue vaccine-exacerbated disease [30].

Antibody Levels, CYD-TDV Vaccine Protection, and Risk of Severe Disease

It is important to note that the development of antibody-dependent enhancement might depend on anti-DENV antibodies' concentrations, and different levels could lead to different outcomes. It was previously shown that although intermediate levels of antibodies can exacerbate disease in a second infection, high antibody levels are protective against severe disease [31]. CYD-TDV may have protected vaccinated individuals against severe dengue and DHF/DSS at the beginning of the trials (CYD14, CYD15, and CYD23). These trials had a follow-up period of two years, and two showed significant protective efficacy against severe dengue [16-18]. Subsequent studies have shown that in participants of the trials, antibody titers were at a maximum after the third dose of the vaccine. Levels then decreased gradually over two years post vaccination and stabilized or slightly increased in year 3. High antibody titers post vaccination may have had a protective effect, explaining the results reported by the trials CYD14 and CYD15 two years after the third dose. Moreover, there were significantly higher levels of antibodies in seropositive individuals compared to the seronegative group, irrespective of age. Seropositive individuals also had a less significant decrease in their titers compared to seronegative [32].

We suspect that in participants with negative serostatus at baseline, the vaccine conferred protection mostly in the first two years. Still, when anti-DENV antibody concentrations started decreasing in subsequent years, levels fell to a range that was not protective anymore, even increasing their risk of severe disease. In participants who were seropositive at baseline, higher anti-DENV antibody concentrations at the beginning of the studies and lesser decreasing titers offered protection in the beginning. They remained high enough to keep conferring protection in the subsequent years of follow-up while never falling in the intermediate-level range, therefore not increasing the risk of severe disease.

Our hypothesis correlates the results of the trials CYD14 and CYD15, in which a significant efficacy was noted at two years, and the findings made by Arredondo-García et al. They showed a higher relative risk of clinically severe hospitalized VCD in years 3-4 than in years 1-2 for all age groups [16,17,24]. Forrat et al. showed higher antibody levels in children nine years and older compared to children younger than nine, which could partly explain why CYD-TDV was more protective against severe dengue in children above nine years than in younger children [25]. We would like to highlight that additional efficacy and safety studies in vaccine receivers aged six to eight years need to be realized since this population might also benefit from the vaccine. In addition, our hypothesis regarding the link between dengue antibody levels, decreasing immunity, and antibody-enhanced disease might benefit from additional investigations to finally elucidate it.

Sabchareon et al. (CYD23) and its long-term safety follow-up by Limkittikul et al. (CYD57) reported severe dengue and hospitalized dengue cases, respectively. Both studies did not show significant differences in the burden of cases in the treatment group compared to the placebo group [18,19]. Therefore, no conclusion could be made from these studies. This absence of difference could, in part, be due to the age of the populations studied. Children enrolled in the trials CYD23 and CYD57 were aged four to 11 years; as we saw previously, the vaccine tends to be more efficient against severe diseases in children aged nine years. The majority of participants of these trials were younger than nine years, which is below the efficacy age range. Consequently, there were fewer subjects in which the vaccine was efficient compared to the trials CYD14 and CYD15, which evaluated older children; therefore, a proportionally higher number of severe cases was found, and they failed to show vaccine efficacy [18,19]. Lanata et al. showed a higher number of dengue cases in the placebo group than in the vaccine group but did not report any cases of severe dengue or DHF; therefore, we could not use its data to help in making our conclusion [21].

TAK-003: A Promising Alternative

Tricou et al. and Biswal et al. evaluated several aspects of Takeda's new tetravalent dengue vaccine. Tricou et al. assessed the safety and immunogenicity of TAK-003 and showed efficacy against non-severe dengue. However, they did not report any cases of severe dengue or DHF [22]. Biswal et al. assessed the efficacy of the TAK-003 vaccine in phase 3 clinical trial in 2020. They divided dengue cases into DHF cases and severe dengue cases based on the two dengue severity classifications of 1997 and 2009. Based on the classification of 1997, the vaccine was shown to have an efficacy of 85.9% against DHF, which was significantly high. However, based on the classification of 2009, they showed that there were more cases of severe dengue in the vaccine group than in the control group. There were overlaps between cases when differentiating them according to the two classifications, with some cases meeting the criteria for both. Consequently, we combined all data, which eventually led to a majority of cases of severe dengue and DHF in the control group compared to the vaccine group [20].

Overall results have shown that the TAK-003 vaccine was efficient against severe dengue and DHF. Moreover, TAK-003 showed good efficacy in all age groups and in dengue seronegative and seropositive individuals, which are major advantages compared to Dengvaxia®. In addition, study conductors reported no safety concerns, and there was no mention of a risk of vaccine-enhanced diseased. Although these are promising results, the follow-up of patients was done over 18 months [20]. As we previously saw with the CYD-TDV efficacy trials, this study duration seems too short to conclude. Longer studies might reveal potential safety issues and long-term adverse events not detectable after less than two years. Nevertheless, long-term follow-up analyses of the TAK-003 vaccine need to be realized and will permit clarification of those aspects, possibly confirming its efficacy and safety. In the future, Takeda's vaccine could become a competitive alternative to CYD-TDV, especially in dengue-seronegative patients and children less than nine years.

Limitations

When writing this systematic review, we wanted to be unbiased in our investigation and come to our conclusions. We then chose to conduct this review only using original and follow-up studies but excluding review articles and secondary analyses. We may have missed additional relevant information to our study by doing so.

We found a satisfying amount of information concerning the vaccine CYD-TDV. However, we only identified two studies about the TAK-003 vaccine, one about vaccine efficacy and one mentioning severe cases. To conclude with one article was challenging, especially because of the duration of the study and the lack of long-term follow-up of participants. We also could not find articles evaluating the vaccines TV003 and TV005 in children; therefore, we could not include them in our review, and we did not get the opportunity to compare them with the other vaccines.

Children (0-18 years) were the population of interest in this study. We aimed to study children because they are at particular risk of developing severe diseases. Therefore, we did not analyze adult subjects. However, adults are also affected by dengue and its severe forms, and the need for a review of vaccine efficacy in populations above 18 years is still relevant. We chose only to include articles in English and excluded articles written in other languages. Where dengue is endemic, many regions are non-English speaking, and articles written by physicians from these areas not published in English could have been useful to us.

## Conclusions

This study aimed to determine if the dengue vaccines were efficient at preventing severe disease in populations under 18 years. We found that CYD-TDV and TAK-003 protect against severe dengue and DHF. Our study demonstrated that one or three doses of the CYD-TDV vaccine confer protection against severe dengue. CYD-TDV showed efficacy against severe disease in dengue-seropositive children older than nine years. We saw that this could be due to vaccine-induced higher antibody concentrations in this age range. The vaccine also demonstrated effectiveness in children aged six to eight years, and we suggested that individuals of these ages could also benefit from it and emphasized that more studies should be realized in this population. We proposed that lower antibodies at baseline and more rapidly decreasing titers could be at the origin of severe disease occurrence in vaccinated dengue-naïve populations, enhancing the need for appropriate alternatives in these subjects.

The TAK-003 vaccine demonstrated satisfying results in terms of efficacy, even in seronegative children. We were limited by the short follow-up time and the low number of studies. We highlighted the importance of observing vaccine receivers for longer periods and continuing studies on this vaccine. In the future, TAK-003 could become a serious alternative to CYD-TDV, particularly in younger and dengue-naïve children. Dengue remains a major health concern today, its prevention in populations living in endemic regions is essential, and research about all preventive strategies needs to be pursued.
